# Stripe-like nanoscale structural phase separation in superconducting BaPb_1−*x*_Bi_*x*_O_3_

**DOI:** 10.1038/ncomms9231

**Published:** 2015-09-16

**Authors:** P. Giraldo-Gallo, Y. Zhang, C. Parra, H.C. Manoharan, M.R. Beasley, T.H. Geballe, M.J. Kramer, I.R. Fisher

**Affiliations:** 1 Geballe Laboratory for Advanced Materials, Stanford University, Stanford, California 94305, USA; 2 Department of Physics, Stanford University, Stanford, California 94305, USA; 3 Ames Laboratory (USDOE), Department of Materials Science and Engineering, Iowa State University, Ames, Iowa 50011-3020, USA; 4 Beijing National Laboratory for Condensed Matter Physics, Institute of Physics, Chinese Academy of Sciences, Beijing 100190, China; 5 Stanford Institute for Materials and Energy Sciences, SLAC National Accelerator Laboratory, Menlo Park, California 94025, USA; 6 Departmento de Física, Universidad Técnica Federico Santa María, Valparaíso, Chile; 7 Department of Applied Physics, Stanford University, Stanford, California 94305, USA

## Abstract

The phase diagram of BaPb_1−*x*_Bi_*x*_O_3_ exhibits a superconducting dome in the proximity of a charge density wave phase. For the superconducting compositions, the material coexists as two structural polymorphs. Here we show, via high-resolution transmission electron microscopy, that the structural dimorphism is accommodated in the form of partially disordered nanoscale stripes. Identification of the morphology of the nanoscale structural phase separation enables determination of the associated length scales, which we compare with the Ginzburg–Landau coherence length. We find that the maximum *T*_c_ occurs when the superconducting coherence length matches the width of the partially disordered stripes, implying a connection between the structural phase separation and the shape of the superconducting dome.

## Abstract (web summary)

In BaPb_1−*x*_Bi_*x*_O_3_ a superconducting dome emerges in the proximity to a charge density wave phase. Here, the authors show with transmission electron microscopy measurements that for superconducting compositions of this allow, the structural dimorphism is accommodated in partially disordered nanoscale stripes.

## Graphical abstract

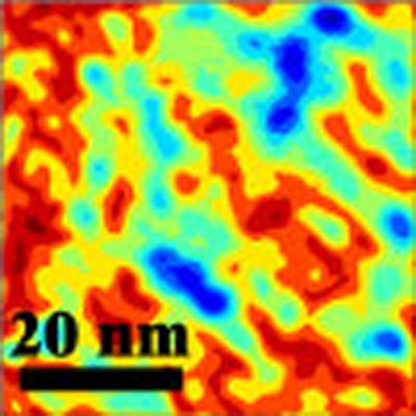

High temperature superconductors are complex materials in which spin, charge, orbital and structural degrees of freedom all appear to play an important role in shaping the emergent electronic properties. Phase segregation in the form of spin stripes^1^, charge stripes^2^,^3^, charge density wave (CDW) nanodomains^4^, lattice modulations^5^,^6^,^7^ and self-organization of dopants^8^,^9^, among others, have been reported for these materials. Different perspectives have been proposed to explain how these phenomena potentially affect important physical properties in these materials, including the superconducting critical temperature *T*_c_ (refs 10, 11, 12, 13). However, many of the details of the nanostructure associated with these subtle forms of phase segregation, and their effects, for example in shaping the superconducting ‘dome', remain enigmatic^11^,^12^,^14^,^15^, partly due to the fact that highly sensitive local probes that have been developed in recent years have only been applied to a small fraction of the materials of interest^7^,^8^,^16^,^17^. For systems with such a variety of interactions, tracking the influence of each individual degree of freedom on the phase separation and on the determination of the electronic properties is challenging. For this reason, the study of simpler superconducting systems can provide useful insights for understanding more complex materials. A model system for the study of how superconductivity is influenced by local CDW instabilities and structural phase separation can be found in the bismuthate superconductors.

The family of bismuthate superconductors results from replacing K for Ba, or Pb for Bi, in BaBiO_3_, a charge disproportionated CDW (CD-CDW) insulator^18^,^19^,^20^,^21^,^22^. This family of superconductors has no magnetic degrees of freedom. On doping, the insulating CDW phase disappears, giving rise to a metallic phase, where superconductivity appears at (maximum) temperatures below 30 and 11 K for K-doping and Pb-doping, respectively^23^,^24^. For the case of BaPb_1–*x*_Bi_*x*_O_3_, superconducting compositions are found to be dimorphic^25^. The nature of the associated structural phase separation, and its effect on the superconducting properties, has, however, not previously been addressed.

BaPb_1–*x*_Bi_*x*_O_3_ has a distorted perovskite (ABO_3_) crystal structure. For the highest Bi concentrations the material comprises two distinct Bi sites, with different Bi–O bond lengths. The origin of the associated CDW has been widely debated^19^,^22^,^26^,^27^. For *x*≤0.8 the average structure comprises a single Bi/Pb site^28^, though EXAFS measurements reveal two distinct Bi–O bond lengths down to at least *x*∼0.25 (refs 29, 30), implying a persistence of the CDW at a local level. Significantly, for all compositions, the perovskite structure is also distorted by rotational instabilities of the oxygen octahedra, which can be described using Glazer's notation^31^,^32^ (see Supplementary Note 1 for an explanation of Glazer's notation). For the insulating end-member compound BaBiO_3_ (*x*=1), and down to *x*=0.9, the unit cell space group is monoclinic *I2/m* (coming from a *a*^0^*b*^−^*c*^−^ tilt, in Glazer's notation); for the metallic end-member compound BaPbO_3_ (*x*=0) and up to *x*≈0.15, and again for 0.35<*x*<0.9, the unit cell space group is orthorhombic *Ibmm* (coming from a *a*^0^*b*^−^*b*^−^ tilt, as shown in Fig. 1a); however, for the region of 0.15<*x*<0.35, which is also the range of compositions for which the material is superconducting, the material is polymorphic, with a fraction of its volume with orthorhombic *Ibmm* symmetry and the rest with tetragonal *I4/mcm* symmetry (coming from a *a*^0^*a*^0^*c*^−^ tilt)^25^. The superconducting volume fraction peaks at the same Bi composition where the tetragonal-to-orthorhombic ratio is maximum, leading to the conclusion that the tetragonal polymorph is the one responsible for superconductivity in this material^25^,^28^. This Bi composition is also the one for which the material has the maximum *T*_c_, that is, the optimal doping composition.

In this article we report the observation of stripe-like structural phase separation in superconducting BaPb_1–*x*_Bi_*x*_O_3_ for compositions spanning optimal doping. We determine the morphology and characteristic length scales of the nanoscale phase separation, revealing intriguing parallels to structural features found in, at least some, cuprate high temperature superconductor materials. We find that the maximum *T*_c_ occurs when the superconducting coherence length matches the width of the partially disordered stripes, implying a connection between the structural phase separation, enhanced coulomb effects due to disorder (localization), inhomogeneous superconducting properties and the shape of the superconducting ‘dome'.

## Results

### Structural phase separation in single crystals

**Figure 1:**
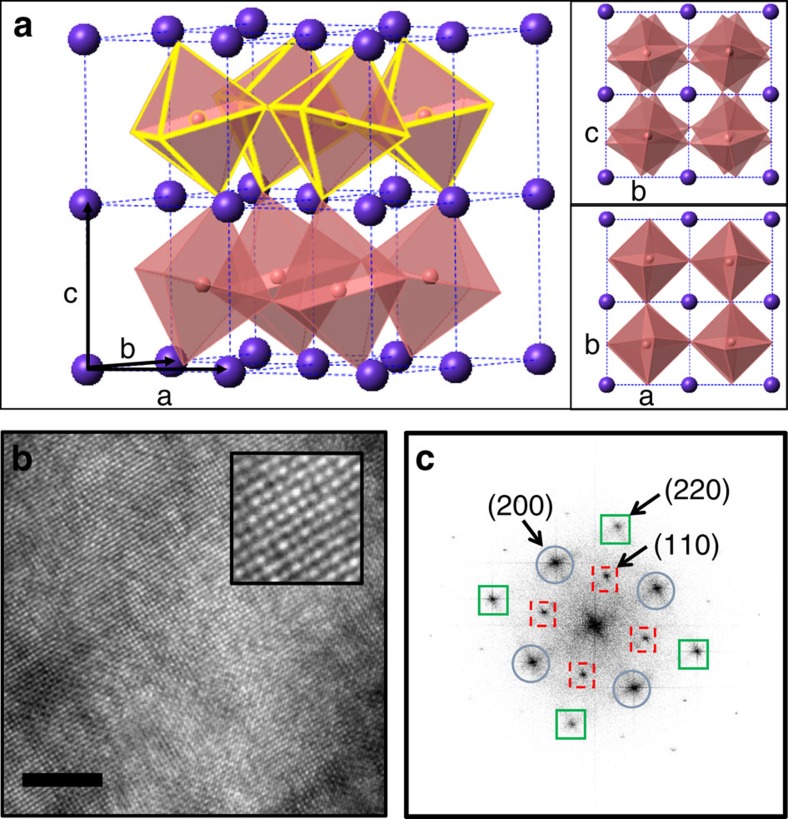
Crystalline structure and HRTEM image. (**a**) Schematic diagram illustrating the *a*^0^*b*^−^*b*^−^ distortion of the cubic perovskite structure, resulting in a orthorhombic *Ibmm* unit cell. The rotations of the octahedra have been exaggerated in the illustrations to more clearly reveal the distortion. (**b**) HRTEM image for a sample with *x*=0.18, looking down the [001] zone axis, revealing a well-ordered structure with coherent planes of atoms. Scale bar is 5 nm long. Inset shows an expanded view of a region of 3 × 3 nm^2^. (**c**) FFT of the image in (**b**), showing peaks corresponding to the {200} (blue-solid circles), {110} (red-dashed squares) and {220} (green-solid squares) sets of reflections. Symbols with solid edges correspond to reflections allowed in both *Ibmm* and *I4/mcm* space groups, while those with dashed edges correspond to reflections forbidden in the *I4/mcm* space group, but allowed in the *Ibmm*.

To investigate how the structural polymorphism is accommodated microscopically in a ‘single crystal' of BaPb_1–*x*_Bi_*x*_O_3_, and its possible consequences for the observed transport and, more interestingly, superconducting properties, high-resolution transmission electron microscopy (HRTEM) measurements were taken for samples with bismuth compositions below, at and above optimal doping. All the HRTEM images taken for all the different compositions reveal a well-ordered structure, as can be observed in the representative 24.1 × 24.1 nm^2^ image in Fig. 1b for a sample with Bi composition of *x*=0.18, and better appreciated in the 3 × 3 nm^2^ expanded view in the inset to this figure. Figure 1c shows its corresponding fast Fourier transform, revealing peaks from both tetragonal (hkl even) and orthorhombic (hkl even and odd, in the tetragonal notation) phases (see Supplementary Tables 1 and 2, and Supplementary Note 2 for more detail).

**Figure 2:**
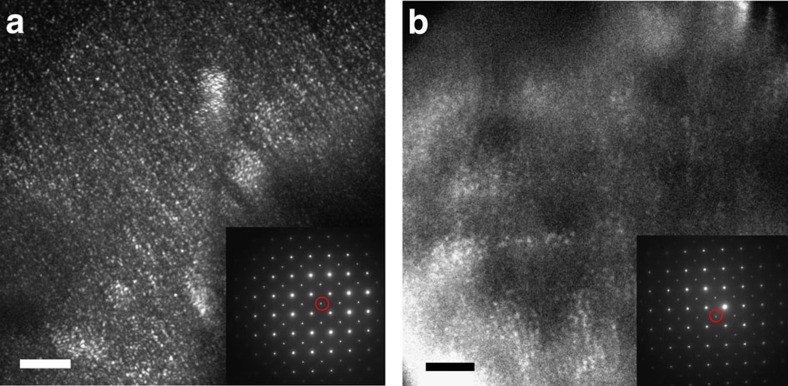
Dark field transmission electron microscopy images. 80.8 × 80.8 nm^2^ dark-field transmission electron microscopy images, as looking down the [001]_T_ zone axis, obtained using the (110) reflection (shown in red in the insets to both figures) for samples with Bi concentration of (**a**) *x*=0.18 and (**b**) *x*=0.28. The scale bars in both figures are 10 nm long. For both images, a patchwork of coherent domains is seen, with stripe-like features, running from top left to bottom right. The stripe-like features are more clearly seen in the filtered-and-reconstructed images, as described in the main text, and shown in Fig. 3 and Fig. 4.

Figure 2 shows dark field (DF) transmission electron microscopy images for samples with Bi concentration of *x*=0.18 and 0.28, along the [001]_T_ zone axis. These DF images were obtained using the orthorhombic (110) reflection shown in the red circle in the insets to their respective figures. For both figures, the size and distribution of the bright regions show a patchwork of coherent domains, with characteristic sizes of 5–10 nm. Indications of a stripy pattern are seen in both images, which for Fig. 2a run from top left to bottom right. This stripy pattern is better identified when recreating a virtual DF image by performing an inverse fast Fourier transform (IFFT) of all the four {110}_T_ reflections. As we will show, by establishing the ‘shape' of the nanoscale phase separation, we are able to determine the associated length scales with better precision than if we assumed an isotropic morphology. This ultimately allows us to make a meaningful comparison with other important characteristic length scales in the material, including the Ginzburg–Landau superconducting coherence length.

**Figure 3:**
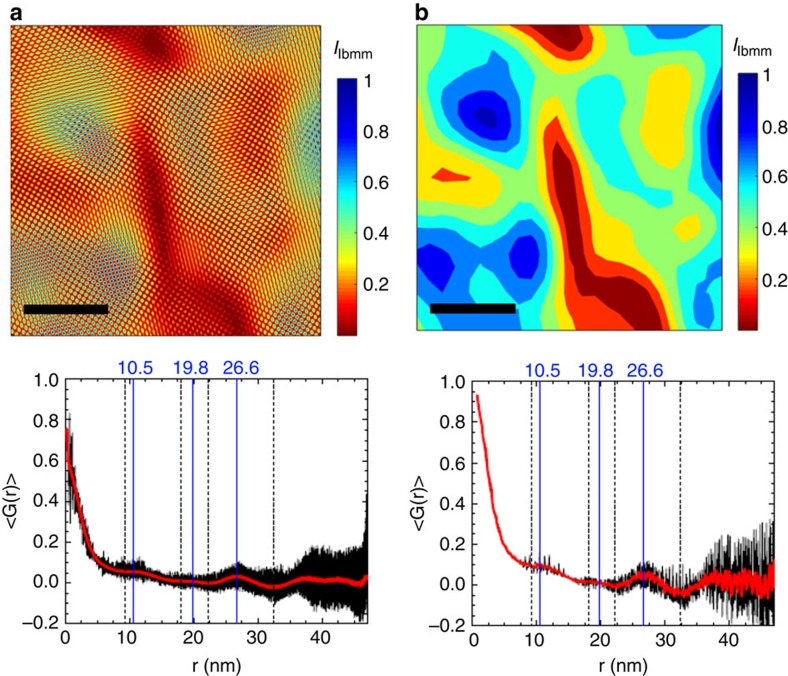
Effect of averaging out the atomic resolution information. (**a**) 19 × 19 nm^2^ portion of a {110}_T_ filtered-and-reconstructed HRTEM image for a sample with bismuth concentration of *x*=0.18, and its corresponding correlation function below it, showing the atomic resolution detail. The scale bar in the image corresponds to 5 nm. Colour scale shows relative normalized intensities: blue regions are more strongly orthorhombic, and red regions are more strongly tetragonal. Dashed-black vertical lines in the correlation function correspond to local minima, and solid-blue vertical lines correspond to local maxima. The exact positions of the maxima are indicated by the numbers in blue above each line. (**b**) Same image as in (**a**), and its corresponding correlation function, after a 7.5 Å × 7.5 Å averaging, eliminating the atomic resolution information while maintaining the broader orthorhombic structural variation. The red curves in the correlation functions are 3 nm smoothing of the original black curves. The average and smoothing procedures do not affect the identification of maxima and minima in the correlation function.

Figure 3a shows a 19 × 19 nm^2^ portion of the {110}_T_ filtered IFFT of the HRTEM image in Fig. 1b. This image keeps the information of both the atomic periodicity as well as a larger-scale contrast variation, reflecting variations in the local ‘orthorhombicity' across the sample. The image in Fig. 3b is the result of a resolution reduction by adjacent averaging, of the image in Fig. 3a, from 0.47 to 7.5 Å per pixel, therefore eliminating the atomic resolution information while keeping the longer range variation in ‘orthorhombicity'. The bottom parts of Fig. 3a,b shows the computed average spatial correlation functions 〈*G*(**r**)〉 of their respective images on top. The vertical lines in 〈*G*(**r**)〉 label local minima and maxima positions |**r**|, being equivalent for both, the original resolution image in Fig. 3a, and the reduced resolution image in Fig. 3b. For the purpose of our analysis, we consider only the reduced resolution images, given that these conserve the information of the longer scale structural variation while reducing the computational requirements.

### Morphology and length scales of structural phase separation

**Figure 4:**
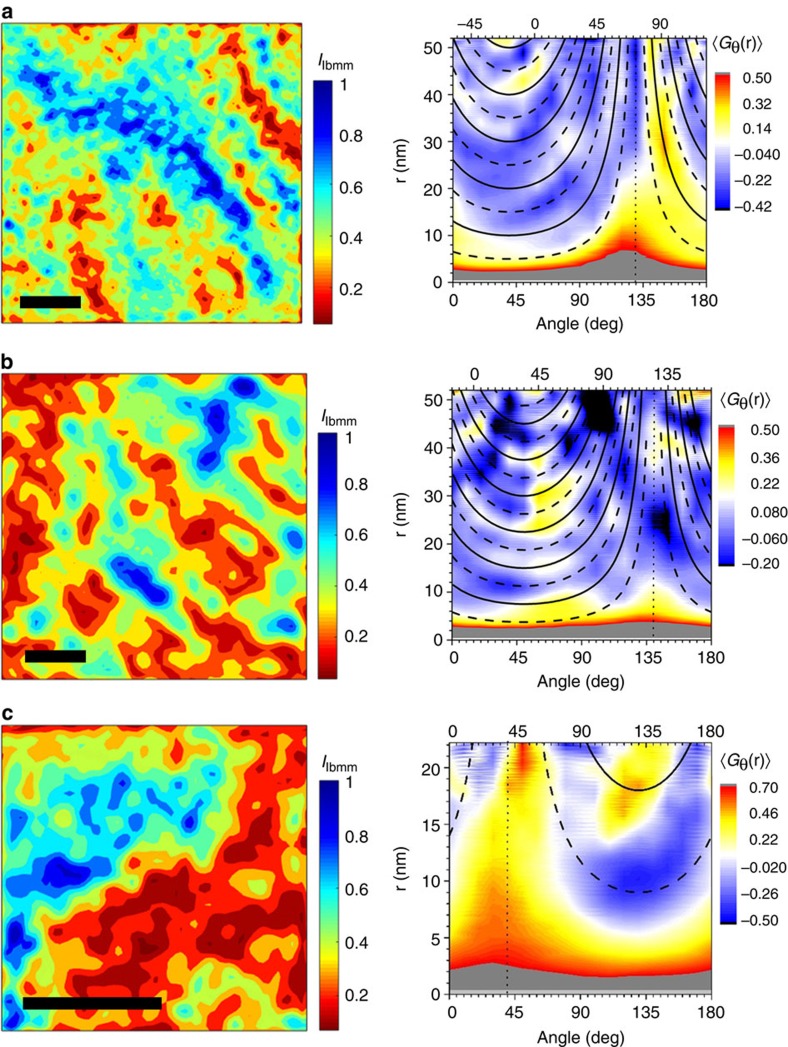
Filtered-and-reconstructed HRTEM images and their correlation function. {110}/{101}_T_ filtered-and-reconstructed HRTEM images (after averaging-out the atomic-scale variation), and their respective angle-dependent correlation functions for BaPb_1–*x*_Bi_*x*_O_3_ samples with bismuth compositions of (**a**) *x*=0.18 (that is, *x*<*x*_opt_, *T*_c_≈7 K) (**b**) *x*=0.24 (*x*∼*x*_opt_, *T*_c_≈10.5 K) and (**c**) *x*=0.28 (*x*>*x*_opt_, *T*_c_≈7 K). The scale bars in all images correspond to 10 nm. Vertical bars next to each image show the normalized intensity colour scale. Blue regions are more strongly orthorhombic, red regions are less strongly orthorhombic (that is, more strongly tetragonal). The colour scale on the right hand side plots represent the value of 〈*G*(**r**)〉. This quantity is plotted as a function of |**r**| (vertical axis) and the angle *θ* with the horizontal (bottom axis) or the [200]_T_ crystalline axis (top axis). The angle-dependent correlation functions reveal clear arcs of intensity, associated with the partially disordered stripe-like nanostructure. Solid and dashed lines represent the best fits to *N* × *d*/cos((*α*–90°)–*θ*) and (2*N*–1) × *w*/cos((*α*–90°)–*θ*) for the local maxima and minima, respectively, as described in the main text. These stripes can be observed in the images in (**a**) and (**b**) running from top left to bottom right.

**Figure 5:**
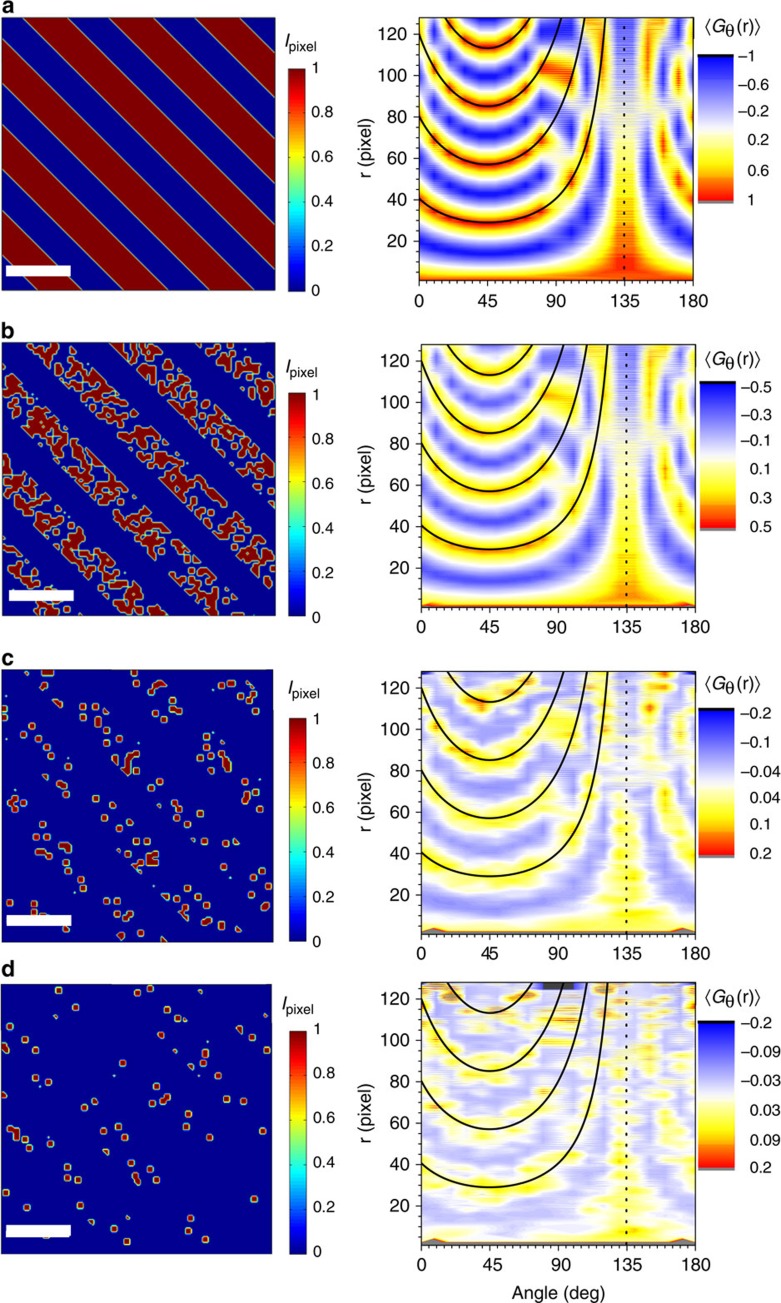
Correlation function for a system with partially disordered stripes. Simulation showing the angle-dependent correlation function 〈*G*_θ_(**r**)〉 for a system with partially disordered stripes. The simulated images have a size of 128 × 128 pixels, with stripes of width *w*=14.1 pixels, separated between them by *d*=28.3 pixels, and running along 135° from the horizontal. The scale bar for all images correspond to 30 pixels. For the different images, a broken-up character of a different level was introduced, as a number of islands of size 3 × 3 pixels, placed at random positions within the red stripes. Each image is characterized by a filling fraction *f*, from 0 (empty stripe) to 1 (full stripe). The filling fraction *f* for each image is: (**a**) *f*=1 (perfect stripe formation), (**b**) *f*=0.5, (**c**) *f*=0.1 and (**d**) *f*=0.05. For each image, the angle-dependent correlation function 〈*G*(**r**)〉 is shown in the right hand panel, and its value is represented in the colour scale, which has different limits for each image. Black lines in these plots follow the functional form N*d/cos((*α*–90°)–*θ*), where *N*=1,2,3,…, *d*=28.3 and *α*=135°. These simulations illustrate how powerful this statistical technique is in revealing weak- or imperfect-stripes formation. As observed in panel (**d**), the stripe formation can be missed at first glance; however, 〈*G*_θ_(**r**)〉 clearly reveals the two-fold symmetry of this image, as well as the periodicity associated with it.

To quantify the length scales associated with the orthorhombic variation, the average spatial correlation function 〈*G*(**r**)〉 and the angle-dependent spatial correlation function 〈*G*_θ_(**r**)〉 were computed for each {110}_T_/{101}_T_ filtered IFFT image (see Supplementary Note 3 for definitions). Figure 4 shows filtered-and-reconstructed HRTEM images for a representative sample of each Bi composition studied (left panels), after a resolution reduction that averages out the atomic-scale information. Both 〈*G*(**r**)〉 (shown in Supplementary Figs 1–3 and Supplementary Note 4) and 〈*G*_θ_(**r**)〉 (shown on the right panels of Fig. 4) of all the images shown reveal local minima and maxima, implying the presence of characteristic length scales for the phase separation. Furthermore, the angular dependent correlation function 〈*G*_θ_(**r**)〉 clearly reveals that there is a particular spatial pattern associated with the phase separation. Inspection of these quantities, in the right hand panels of Fig. 4, reveals arcs of intensity with an approximately two-fold rotational symmetry. The arcs are imperfect, but repeat with a fixed periodicity, implying a self-organized pattern of phase separation over remarkably large length scales. Such a pattern of intensity in 〈*G*_θ_(**r**)〉 is consistent with a real space phase separation comprising partially disordered stripes (see Fig. 5, and Supplementary Note 5). For a system with stripes separated by a distance *d* and running along an angle *α* with respect to the horizontal, the distance between stripes as measured at an angle *θ* is given by *N* × *d*/cos((*α*–90°)–*θ*) (with *N*=1,2,3,…), which diverges at *θ*=*α*. As can be observed in Fig. 4 (and in similar data shown in Supplementary Figs 1–3), most of the samples studied exhibit this characteristic dependence, with periodic maxima (shown by solid lines in the figure) and minima (dashed lines) that approximately follow such an inverse cosine function. The orientation of the stripes with respect to the crystal axes is not identical for all images studied, but on average it is close to 29°±22° from the [100]_T_ orientation (see Supplementary Fig. 4 and Supplementary Note 6). These stripes are clearly evident in the larger area real space images shown in the left hand panels of Fig. 4a,b, running approximately top left to bottom right. In addition to the separation of stripes, inspection of the images in Fig. 4 reveals that there is a shorter (and more isotropic) length scale of structural variation, which describes the broken-up character of the stripes. This length scale can be seen more clearly in the average correlation function as a kink in the low-r tail, which can be better identified in the derivative of 〈*G*(r)〉. (see Supplementary Fig. 5 and Supplementary Note 7). Differences in the IFFT images reconstructed using different set of orthorhombic reflections, (110)_T_ or 
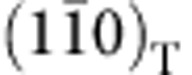
, suggests that there are more complicated components to this phase separation, involving subtle tilts with respect to the average crystal axes (see Supplementary Figs 6–8 and Supplementary Note 8). However, given that we are interested in the average variation of orthorhombicity across areas of the crystals, we restrict our analysis to the filtered IFFT images reconstructed with the set of all four {110}_T_/{101}_T_ (*Ibmm*) reflections, which are sufficient to unambiguously determine the associated length scales.

**Figure 6:**
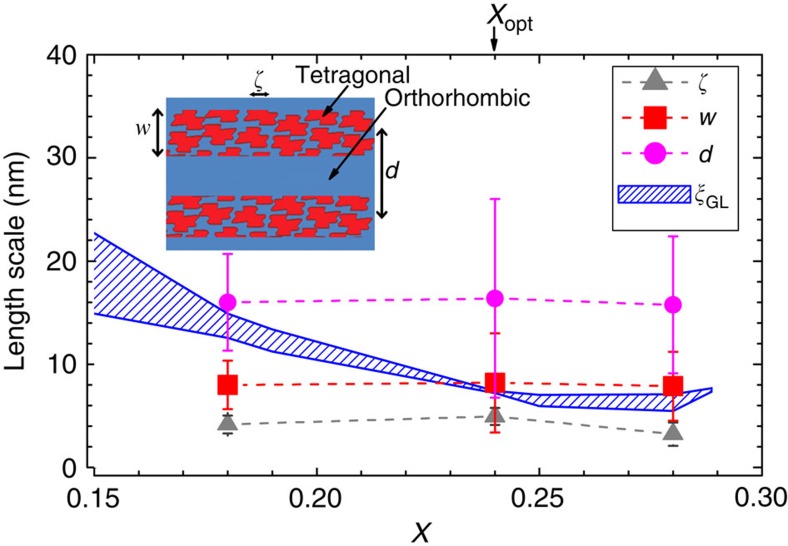
Length scales of phase separation. Average characteristic lengths of phase separation for BaPb_1–*x*_Bi_*x*_O_3_ as a function of *x*. These data are contrasted with the Ginzburg–Landau coherence length *ξ*_GL_(0), represented in the blue curves. Error bars represent the standard deviation from the average of all the samples measured for each composition.

Although the stripe-like character of the structural phase separation is imperfect, nevertheless by identifying the morphology of the nanostructure we are able to define the characteristic length scales of phase separation in terms of three simple parameters (see inset to Fig. 6): the stripe period, *d*, (that is, the distance between stripes of similar ‘orthorhombicity', determined from the maxima of 〈*G*_θ_(**r**)〉); the stripe width, *w* (estimated from the regions of minimum values in 〈*G*_θ_(**r**)〉, that is, stripes half-period, which can be used as a measure of the upper bound to the width of individual stripes); and the length scale associated with disorder within a stripe, *ζ*, (identified in the derivative of the low-r tail of 〈*G*(**r**)〉, as shown in Supplementary Fig. 5 and Supplementary Note 7). The analysis described above, was performed for a total of five *x*=0.18 samples, four *x*=0.24 samples and four *x*=0.28 samples (all of which are shown in the Supplementary Material), and the average value of *d*, *w* and *ζ* for each Bi composition were calculated. The results are summarized in Fig. 6, together with the error obtained by calculating the s.d. from the average value.

## Discussion

**Figure 7:**
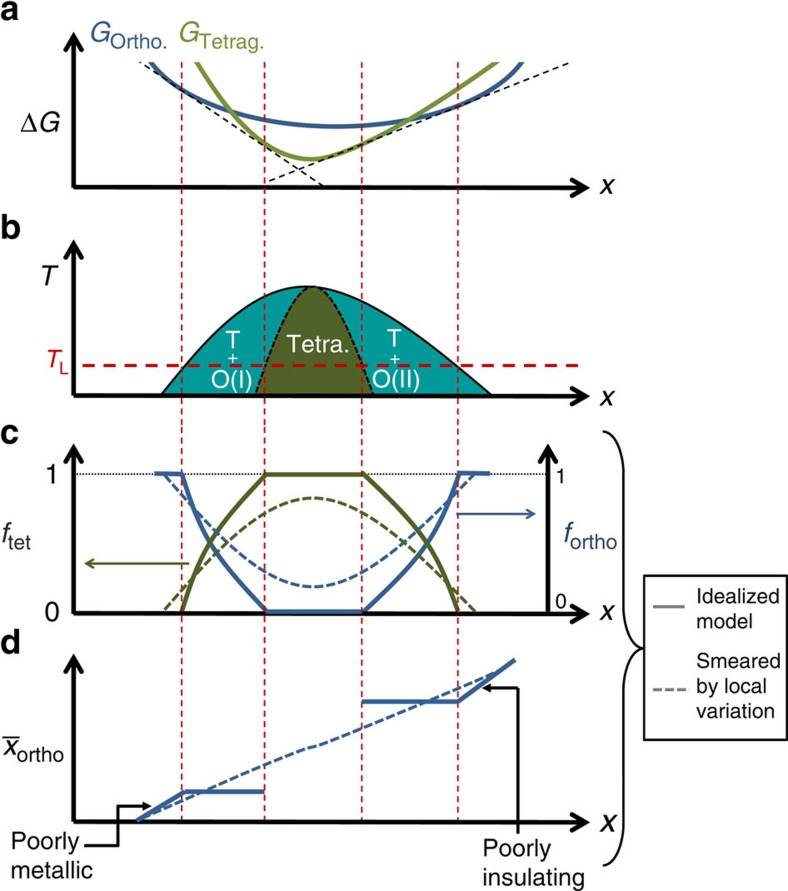
Structural phase separation scenario in BaPb_1–*x*_Bi_*x*_O_3_. (**a**) Schematic diagram of the Gibbs free energy of orthorhombic (*G*_ortho._) and tetragonal (*G*_tetrag._) phases as a function of composition at a given low temperature *T*=*T*_L_. (**b**) The corresponding phase diagram, showing regions of phase coexistence. At *x*=*x*_opt_ the tetragonal fraction is maximum. For *x*<*x*_opt_ the orthorhombic phase is labelled as O(I), and it is a low Bi phase, presumably metallic. For *x*>*x*_opt_ the orthorhombic phase is labelled O(II), and it is a rich-Bi phase, presumably insulating. (**c**) Fraction of tetragonal (*f*_tet_, left axis) and orthorombic (*f*_ortho_, right axis) phases as a function of composition, at temperature *T*=*T*_L_ (marked by the red horizontal line in panel (**b**)). (**d**) Spatial average of orthorhombic composition, 
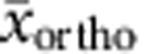
, as a function of composition, for the same temperature *T*=*T*_L_. Solid lines in panels (**c**) and (**d**) show the evolution of these quantities with composition for the ideal case depicted in (**a**), whereas dashed-curves show the evolution for the case where the local free energy is modified by local strain, giving rise to a distribution of free energies that smear the otherwise sharp distinctions and features of the tetragonal and orthorhombic fractions, and spatial average of orthorhombic composition.

The phase separation of tetragonal and orthorhombic polymorphs in BaPb_1–*x*_Bi_*x*_O_3_ is presumably driven by changes in the relative free energy of the two phases, both as a function of temperature and composition^33^. Such a scenario is illustrated schematically in Fig. 7. The resulting morphology is reminiscent of spinodal decomposition, but the physical origin is somewhat different in this case, involving two competing phases. Significantly, in such a scenario, the composition *x*_opt_∼0.24, at which the tetragonal volume fraction is maximal, marks the separatrix between formation of two different orthorhombic phases, both with the same structure, but one with a lower Bi concentration (O(I), for compositions *x*<*x*_opt_), and one with a higher Bi concentration (O(II), for *x*>*x*_opt_). It has been previously shown for various metallic precipitates embedded in metallic matrices (Cu in Al, Ag in Cu and Ag in Al, among others) that inhomogeneous strain can cause local variations in the free energy, modifying phase equilibria^34^. Therefore, it is reasonable to anticipate that the sharp distinctions in composition between the O(I) and O(II) phases will be blurred in practice (Fig. 7c,d). The resulting continuous variation in composition, and presumably lattice parameter, is consistent with results of recent X-ray and neutron diffraction measurements^25^. Considering the temperature dependence of the resistivity for compositions that have only an orthorhombic structure, it is clear that Bi substitution leads to a progressive evolution of the electronic properties of the orthorhombic phase from a ‘bad metal' for *x*<<*x*_opt_ (that is, *dρ*/*dT*>0, but with a very large absolute value of the resistivity) to a ‘bad insulator' for *x*≫*x*_opt_ (that is, *dρ*/*dT*<0, but nevertheless extrapolating to a finite conductivity at *T*=0) (refs 18, 35, 36). It is unclear whether this evolution of the electronic properties of the orthorhombic phase is driven by disorder due to the increasing Bi concentration, or a progressive increase in the CDW correlation length, or indeed a combination of both effects, but tunnelling data clearly indicates that the zero temperature conductivity decreases to zero linearly in the entire range from *x*=0 to *x*=0.3, and that the associated zero bias tunnelling anomaly also varies smoothly over this range^37^. Of particular significance for the following discussion, if the Bi concentration deviates from *x*_opt_∼0.24 in either direction, and the tetragonal volume fraction correspondingly diminishes, the phase separation results in small islands of superconducting tetragonal material with a characteristic length scale embedded in a matrix of orthorhombic BaPb_1–*x*_Bi_*x*_O3 that is either poorly conducting for *x*<*x*_opt_ or poorly insulating for *x*>*x*_opt_. This distinction has important consequences for the evolution of the superconducting properties.

On the basis of the {110}_T_/{101}_T_ filtered-and-reconstructed HRTEM images, averaged orthorhombic intensity distribution histograms can be generated for each Bi concentration (see Supplementary Figs 9–13 and Supplementary Note 9). Analysis of these histograms reveals that the tetragonal volume fraction does indeed peak at the same composition as optimal doping, confirming earlier reports based on X-ray and neutron diffraction experiments in polycrystalline samples^25^, and suggesting a direct connection between the tetragonal distortion and superconductivity.

The significance of the structural modulation with respect to the superconducting properties can be readily appreciated by comparing the associated length scales of the disordered stripes, *d*, *w* and *ζ* (solid data points in Fig. 6), with the Ginzburg–Landau coherence length, *ξ*_GL_(0) (blue curve in the same figure), for samples with *x*<*x*_opt_, *x*≈*x*_opt_ (optimally doped), and *x*>*x*_opt_, with approximate *T*_c_ values of 7, 10.5 and 7 K, respectively. We estimate *ξ*_GL_(0) from *H*_c2_(0) for a series of superconducting compositions, including the ones presented in this paper, having used the standard Werthamer–Helfand–Hohenberg approximation to determine *H*_c2_(0) from *H*_c2_(*T*). We employed both 50 and 90% criteria to extract *H*_c2_(*T*) from resistive transitions, as we previously showed in refs 35 and 36, leading to a narrow band of estimated values for *ξ*_GL_(0). Inspection of Fig. 6 reveals that the three length scales associated with the phase separation are of the same order of magnitude as the superconducting coherence length, and largely independent of Bi concentration. The shortest length scale, *ζ*, which characterizes the size of coherent regions within a given stripe, has a weak composition dependence, but does not grow to be larger than the superconducting coherence length for any composition, and is therefore expected to be less relevant than the larger length scales *d* and *w* associated with the period and width of the stripes. However, this length scale is presumably the one associated to the accommodation of the volume fraction of each polymorph. For low Bi concentrations, *x*<*x*_opt_, the coherence length is larger than the width of individual stripes. However, at optimal doping, the width of individual stripes almost exactly matches the superconducting coherence length. Further increasing the Bi concentration appears to result in a saturation of *ξ*_GL_(0) which remains comparable to *w*. This behaviour is highly suggestive of an important role for the nanostructure in determining the shape of the superconducting dome, as we describe below.

In the context of an electronically inhomogeneous system, where the Coulomb potential seen by electrons varies spatially in a periodic way, with characteristic length *λ*, it has been shown theoretically that *T*_c_ does not necessarily track the pairing scale *Δ*_0_, that is, the superconducting gap magnitude^14^,^38^. Rather, the evolution of *T*_c_ is bounded above by two parameters: the pairing scale *Δ*_0_ and the phase ordering temperature *T*_θ_. In the limit, where *λ*<<*ξ* (where *ξ* is the superconducting coherence length), *T*_θ_≫*Δ*_0_, and *T*_c_ will be determined by *Δ*_0_. However, in the limit *λ*≫*ξ*, the phase ordering temperature *T*_θ_ is small compared with the pairing amplitude *Δ*_0_, and *T*_c_ is entirely determined by *T*_θ_, meaning that *T*_c_ is suppressed with respect to *Δ*_0_. In this regime the material behaves as a granular superconductor, characterized by superconducting ‘islands' that are only weakly coupled. For a system where the length scale of phase separation evolves with respect to the superconducting coherence length (or vice-versa), the maximum *T*_c_ value is obtained in the crossover regime of the curves of *T* and *Δ*_0_, which happens at *λ*∼*ξ*. This regime has been dubbed ‘optimal inhomogeneity'^14^,^39^. Although this model was originally developed based on a single-band Hubbard Hamiltonian in an uniform two-dimensional lattice, for which phase segregation with characteristic length scales of the order of the superconducting coherence length is spontaneous and originates from the strong electronic correlations, the consequences of the phase segregation on the shaping the superconducting dome in BaPb_1–*x*_Bi_*x*_O_3_ are still relevant. For BaPb_1–*x*_Bi_*x*_O_3_, the phase separation is structural and quenched from high temperatures (R.J. Cava, personal communication); however, given the large differences in electronic properties between both polymorphs, the modulation of the pairing interaction in the nanoscale is present and mimics the physical landscape found in the model presented above. Such a bounding of the superconducting dome by *T*_θ_ and *Δ*_0_ has been widely discussed in the field of granular superconductivity^40^,^41^,^42^,^43^. Similarly, the optimization and enhancement of *T*_c_ in heterostructures formed by the alternation of metallic stripes of width *L*∼*λ*_F_, and superconducting stripes of width *W*∼*ξ*_0_, as a consequence of a possible shape resonance has been discussed by several authors^12^,^44^. Such configurations have been observed to naturally occur in cuprate superconductors, such as Bi_2_Sr_2_CaCu_2_O_8+*y*_ and La_2_CuO_4_ (refs 5, 6), and have been proposed to generate an enhancement of *T*_c_.

**Figure 8:**
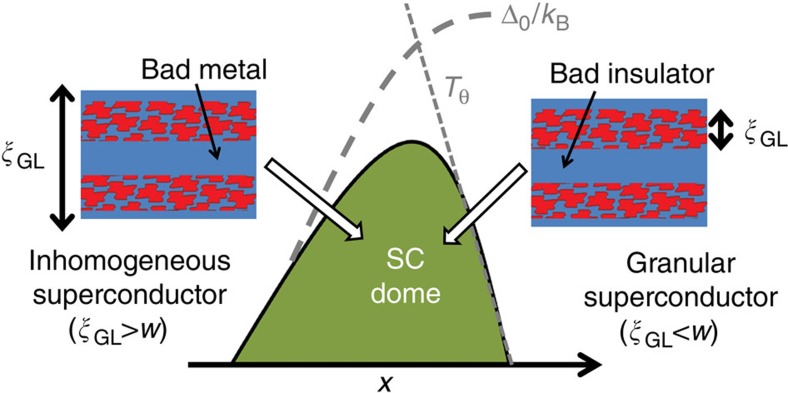
Structural Phase separation and the superconducting dome. Stylized cartoon illustrating the superconducting ‘dome' in the *T*-*x* plane of BaPb_1–*x*_Bi_*x*_O_3_ and the effect of the competing length scales associated with structural phase separation and the superconducting coherence length (see discussion in main text). Red and blue regions correspond to tetragonal and orthorhombic polymorphs respectively, arranged in partially disordered stripes of width w. The pairing interaction is understood to originate in the tetragonal material^25^. In the cartoon, *ξ*_GL_ is the Ginzburg–Landau superconducting coherence length, and *Δ*_0_/*k*_B_ and *T*_θ_ are the pairing amplitude and the phase condensation temperatures, respectively.

The phenomenology of BaPb_1–*x*_Bi_*x*_O_3_ appears to be consistent with a scenario in which the shape of the superconducting ‘dome' is determined by the relative evolution of the pairing amplitude Δ_0_ and the phase ordering temperature *T*_θ_, and in which tetragonal and orthorhombic polymorphs correspond to regions of the bulk material with large and small pairing interactions, respectively. The evolution with doping of the relative length scales characterized by *ξ*_GL_ and the phase separation is very suggestive of optimal doping being a turning point from a macroscopic inhomogeneous superconductor (with *ξ*_GL_ bigger than other characteristic length scales associated with disorder) for *x*<*x*_opt_ to a phase-fluctuation-dominated granular superconductor for *x*>*x*_opt_ (illustrated schematically in Fig. 8). Indeed, several signatures of granular superconductivity are observed in this regime, such as negative magnetoresistance for fields above *H*_c2_(*T*), and scaling reminiscent of a superconductor-insulator quantum phase transition^35^. In addition, scanning tunnelling spectroscopy measurements for compositions beyond optimal doping show a large variation in gap values as a function of position, with maximum values exceeding those found in the higher *T*_c_ optimally doped material (C.P., *et al*. manuscript in preparation), suggesting that samples with *x*>*x*_opt_ have a larger local pairing amplitude than expected for their macroscopic *T*_c_, and even for an 11 K superconductor. This observation is consistent with a macroscopic *T*_c_ being bounded by the phase ordering line, *T*_θ_, that is, with a granular superconductor picture (see Fig. 8). Significantly, these observations imply that for *x*>*x*_opt_ the superconducting phase of this material (the tetragonal polymorph) is in fact a higher-temperature superconductor, possibly even comparable to the other bismuthate superconductor Ba_1–*x*_K_*x*_BiO_3_ (ref. 37).

In the above analysis, the only significance of the stripe-like character of the nanostructure of BaPb_1–*x*_Bi_*x*_O_3_ has been that it has enabled us to establish the characteristic length scales with a little more precision than if we had assumed a more isotropic morphology. However, the stripe-like morphology possibly has a much deeper significance. In the context of BaPb_1–*x*_Bi_*x*_O_3_, this might provide a natural means to understand the unusual scaling behaviour observed at the superconductor-insulator transition close to optimal doping in this material^35^,^36^, motivating theoretical investigation of percolation effects near the quantum phase transition for a material with a ‘stripy' morphology^45^,^46^,^47^,^48^,^49^. More broadly, several families of underdoped cuprates have been shown to exhibit stripe and/or unidirectional CDW formation^1^,^2^,^3^,^4^,^5^,^6^,^7^,^8^,^9^. Complexity and phase separation in these systems is an active field of study^5^,^6^,^7^,^8^,^9^,^12^,^50^,^51^, and the role and importance of these phenomena in the cuprates is still a matter of discussion. Significantly, in both cases, cuprates and bismuthates, the stripe-like phase separation and superconductivity are found to have comparable length scales. In this broader context, BaPb_1–*x*_Bi_*x*_O_3_ provides a model system to explore the effects of inhomogeneity and stripe-like phase separation on superconductivity.

## Methods

### Experimental details

Single crystals of BaPb_1–*x*_Bi_*x*_O_3_ were grown using a self-flux technique, similar to that described in (ref. 52). The cation composition was determined by electron microprobe analysis. These measurements revealed an uniform composition across each sample, within the experimental uncertainty (±0.02).

To obtain HRTEM measurements of BaPb_1–*x*_Bi_*x*_O_3_, samples of each concentration studied were crushed in liquid-nitrogen-cooled ethyl alcohol and the liquid was allowed to warm to room temperature. The slurry was stirred and a small droplet was placed on a holey carbon grid and dried in air. Measurements were taken at room temperature. The samples were analysed using a FEI G2 F20TEM Tecnai STEM operated at 200 keV. Thin areas were analysed with selected area diffraction, energy dispersive spectroscopy and high-resolution imaging. Thin areas were aligned with either the [010] or the [001] zone axis based on indexing to the *Ibmm* structure (space group no. 74), showing clear lattice fringes in the HRTEM. All the HRTEM images taken reveal a well-ordered structure, from which the information about the structural phase separation can be obtained in the way described in the text.

## Additional information

**How to cite this article:** Giraldo-Gallo, P. *et al*. Stripe-like nanoscale structural phase separation in superconducting BaPb_1–*x*_Bi_*x*_O_3_. *Nat. Commun.* 6:8231 doi: 10.1038/ncomms9231 (2015).

## Supplementary Material

Supplementary Information Supplementary Figures 1-13, Supplementary Tables 1-2, Supplementary Notes 1-9 and Supplementary References.
