# Supplementary material for: Stripe-like nanoscale structural phase separation in superconducting BaPb1−xBixO3
Source: Nat Commun. 2015 Sep 16;6:8231. doi: 10.1038/ncomms9231 (PMC4595596; doi:10.1038/ncomms9231)
Supplement: Supplementary Information — Supplementary Figures 1-13, Supplementary Tables 1-2, Supplementary Notes 1-9 and Supplementary References. [file ncomms9231-s1.pdf]

SUPPLEMENTARY FIGURES

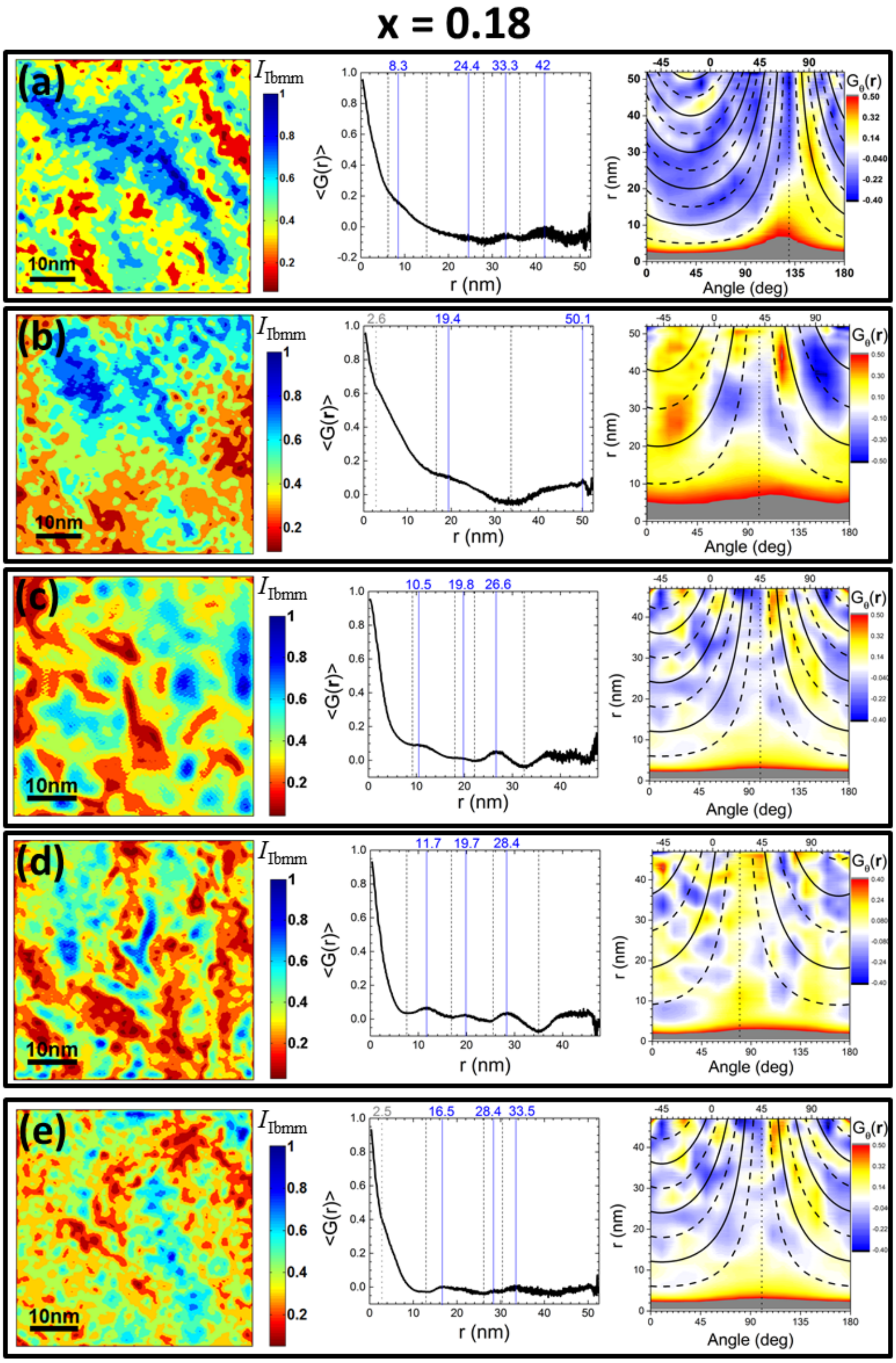

Supplementary Figure 1 – **Filtered HRTEM images and their correlation function for** x**=0.18 samples**. $\{110\}_T/\{101\}_T$ filtered-and-reconstructed HRTEM images (first-column figures), for different samples with bismuth concentration of x=0.18. For each horizontal panel, the center column shows the corresponding average spatial correlation function, $\langle G(\mathbf{r})\rangle$. Solid blue vertical lines indicate the local maxima in $\langle G(\mathbf{r})\rangle$, while dashed-black vertical lines indicate local minima. The third column in each horizontal panel shows the angle-dependent spatial correlation function, $\langle G_\theta(\mathbf{r})\rangle$ on a color scale, as a function of $|\mathbf{r}|$ (vertical axis) and the angle θ with the horizontal (bottom-axis) or the $[200]_T$ crystalline axis (top-axis). Solid and dashed lines represent the best fits to $N \times d/\cos((\alpha - 90^\circ) - \theta)$ and $(2N - 1) \times w/\cos((\alpha - 90^\circ) - \theta)$ for the local maxima and minima respectively.

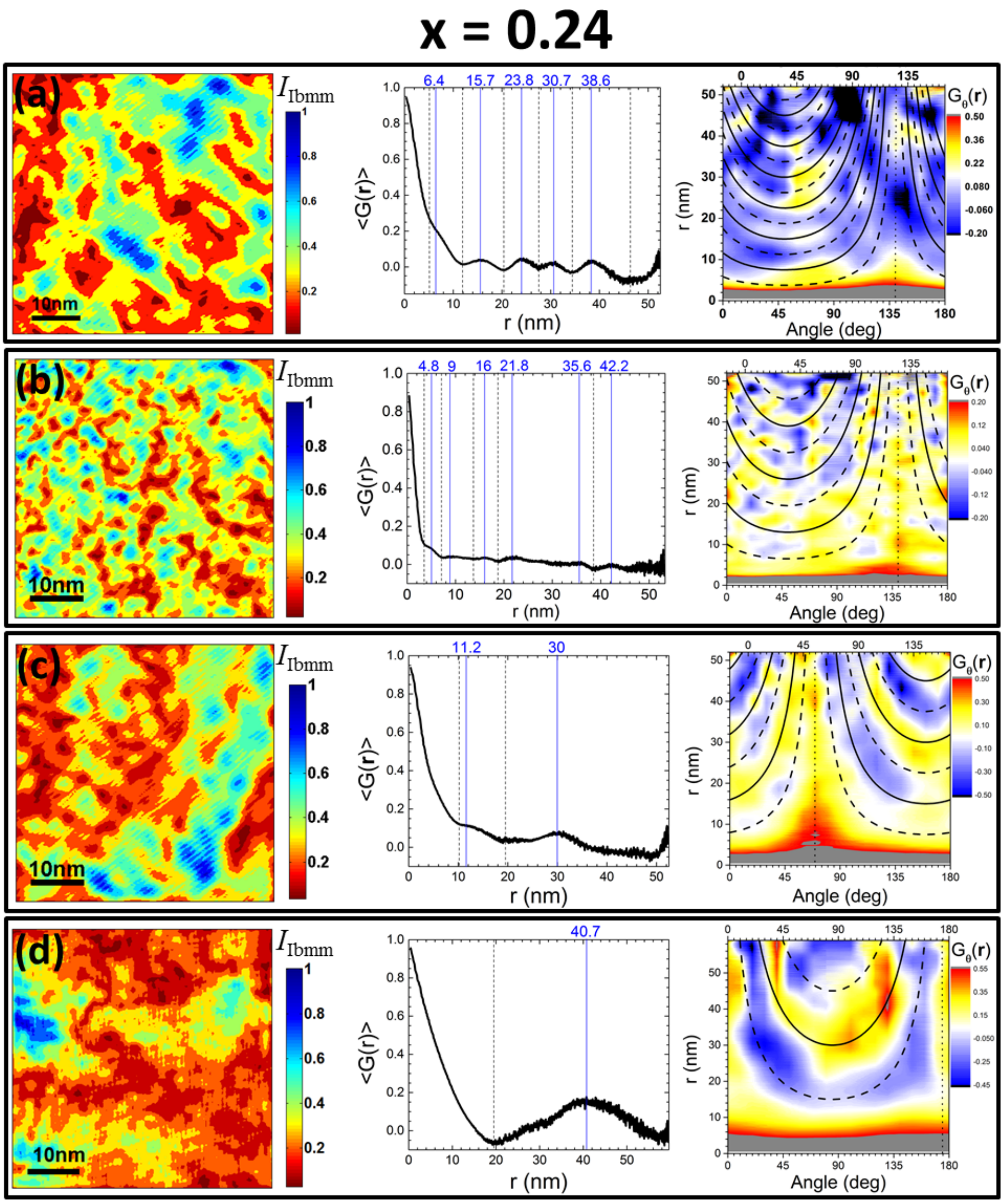

Supplementary Figure 2 – Filtered HRTEM images and their correlation function for x=0.24 samples. $\{110\}_\mathrm{T}/\{101\}_\mathrm{T}$ filtered-and-reconstructed HRTEM images (first-column figures), for different samples with bismuth concentration of x=0.24. For each horizontal panel, the center column shows the corresponding average spatial correlation function, $\langle G(\mathbf{r})\rangle$. Solid blue vertical lines indicate the local maxima in $\langle G(\mathbf{r})\rangle$, while dashed-black vertical lines indicate local minima. The third column in each horizontal panel shows the angle-dependent spatial correlation function, $\langle G_\theta(\mathbf{r})\rangle$ on a color scale, as a function of $|\mathbf{r}|$ (vertical axis) and the angle θ with the horizontal (bottom-axis) or the $[200]_\mathrm{T}$ crystalline axis (top-axis). Solid and dashed lines represent the best fits to $N \times d/\cos((\alpha - 90°) - \theta)$ and $(2N-1) \times w/\cos((\alpha - 90°) - \theta)$ for the local maxima and minima respectively.

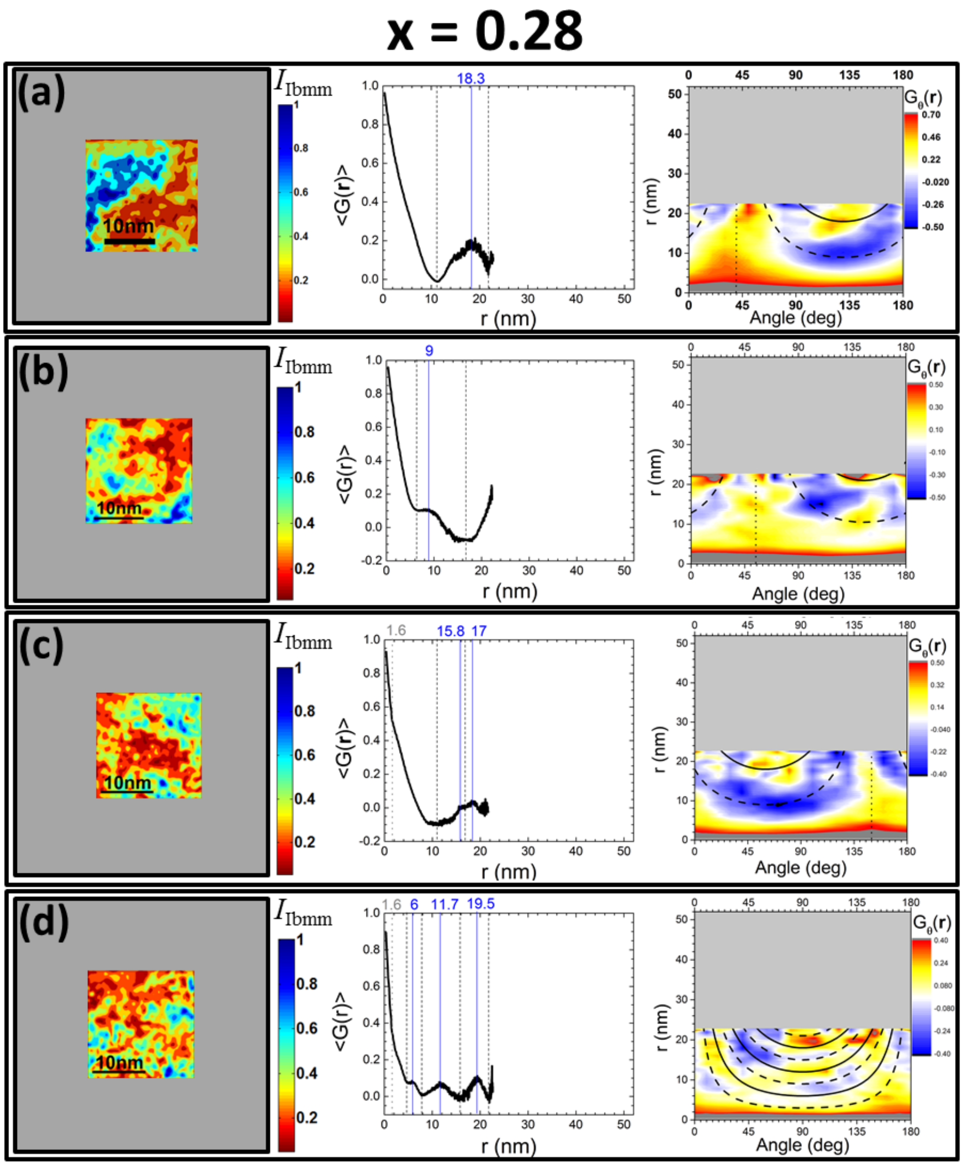

Supplementary Figure 3 – Filtered HRTEM images and their correlation function for x=0.28 samples. $\{110\}_{\mathrm{T}}/\{101\}_{\mathrm{T}}$ filtered-and-reconstructed HRTEM images (first-column figures), for different samples with bismuth concentration of x=0.28. These images were taken for a smaller area than the ones for the x =0.18 and x =0.24 samples. To preserve a direct comparison with those data, the images are shown on the same scale as for figs. 1 and 2. For each horizontal panel, the center column shows the corresponding average spatial correlation function, $\langle G(\mathbf{r})\rangle$. Solid blue vertical lines indicate the local maxima in $\langle G(\mathbf{r})\rangle$, while dashed-black vertical lines indicate local minima. The third column in each horizontal panel shows the angle-dependent spatial correlation function, $\langle G_\theta(\mathbf{r})\rangle$ on a color scale, as a function of $|\mathbf{r}|$ (vertical axis) and the angle θ with the horizontal (bottom-axis) or the $[200]_{\mathrm{T}}$ crystalline axis (top-axis). Solid and dashed lines represent the best fits to $N \times d/\cos((\alpha-90°)-\theta)$ and $(2N-1)\times w/\cos((\alpha-90°)-\theta)$ for the local maxima and minima respectively.

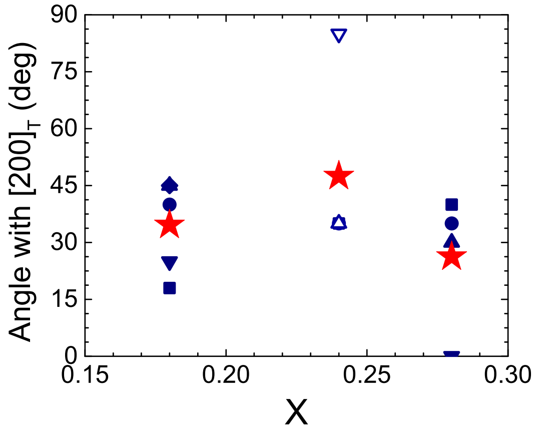

Supplementary Figure 4 – **Orientation of stripes with respect to the** $[200]_T$ **crystalline axis**, as a function of Bi concentration, for all the samples studied. Open blue symbols show the orientation for samples with images taken along the $[001]_T$ zone axis; full blue symbols show the orientation for samples with images taken along the $[010]_T$ zone axis. Star-shape full red symbols show the average orientation for each composition.

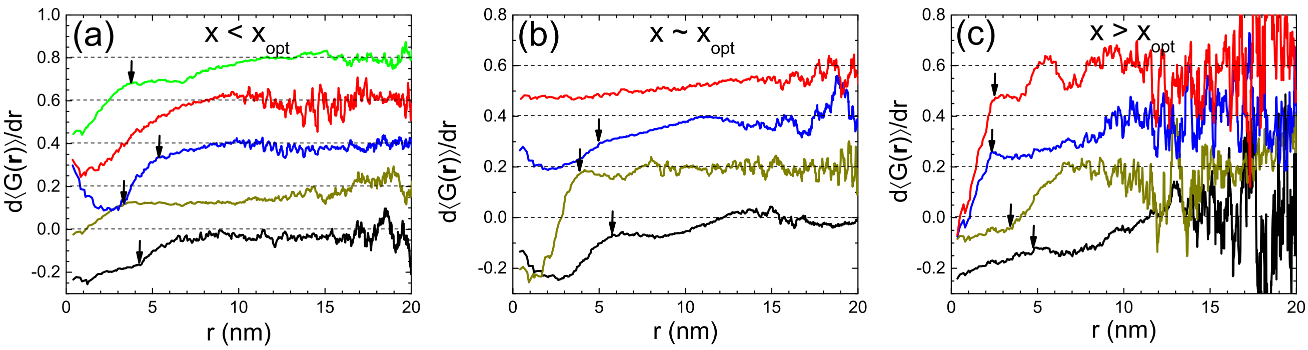

Supplementary Figure 5 – **Derivative of the average correlation function** $\langle G(\mathbf{r})\rangle$ (for the low-r tail region) of different filtered-and-reconstructed images of samples with Bi concentrations of **(a)** x =0.18 ($x < x_{\rm opt}$), **(b)** x =0.24 ($x \approx x_{\rm opt}$) and **(c)** x =0.28 ($x > x_{\rm opt}$). Dashed horizontal lines mark the $d\langle G(\mathbf{r})\rangle/dr = 0$ zone for each curve. Arrows mark the points determining the disorder length-scale within a stripe, ζ. Average values of ζ are shown in Fig. 6 of the main manuscript, as a function of x.

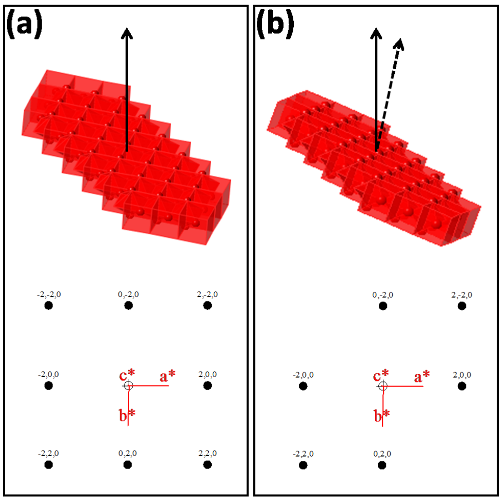

Supplementary Figure 6 – **Effect of zone-axis tilting in the TEM diffraction pattern**. **(a)** Simulated electron diffraction pattern for an ideal cubic lattice with the zone axis aligned with the [001] direction. **(b)** Simulated electron diffraction pattern for the same system, but where the zone axis is slightly tilted with respect to the [100] direction. The effect of this tilting is the disappearance of the (220) reflections, while maintaining the $(2\bar{2}0)$ ones. If the lattice is tilted in the opposite direction, then the peaks that disappear are the $(2\bar{2}0)$ ones. These subtle tiltings across the area of a sample can generate differences in the IFFT-images reconstructed with one or the other set of reflections.

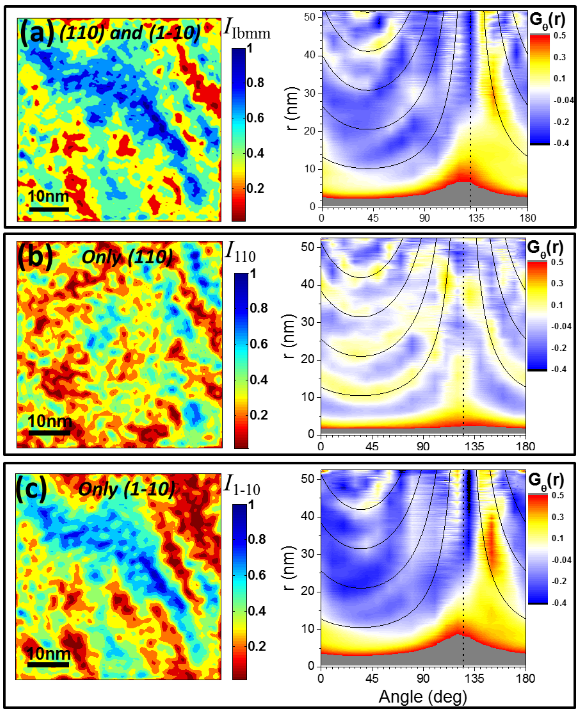

Supplementary Figure 7 – **Four-reflections vs. two-reflections images for** $\boldsymbol{x}$ **=0.18 samples**. Variations in the filtered-and-reconstructed HRTEM images by using four or two *Ibmm* reflections, and their respective angle-dependent correlation functions, for a sample with bismuth concentration of x=0.18. **(a)** Same image than in Fig. 4(a) or fig 1(a), obtained by performing the inverse Fourier transform of the four *Ibmm* reflections, $\{110\}_{\mathrm{T}}$ (formed by the $(110)_{\mathrm{T}}$ and $(1\bar{1}0)_{\mathrm{T}}$ peaks). **(b)** filtered-and-reconstructed image corresponding to the same HRTEM image, but now obtained by only using a set of two reflections corresponding to $(110)_{\mathrm{T}}$ and **(c)** $(1\bar{1}0)_{\mathrm{T}}$. For all of this images, a resolution reduction from 0.51Å per pixel, to 4.1Å per pixel was performed, in order to average-out the atomic scale information. The angle dependent correlation function for each image is shown in the right hand panels, from which the stripe-like angle evolution can be recognized better in the reconstruction using one set of reflections than with the other. The reconstruction resulting from all the four reflections is a combination of the reconstructions of the individual sets of reflections.

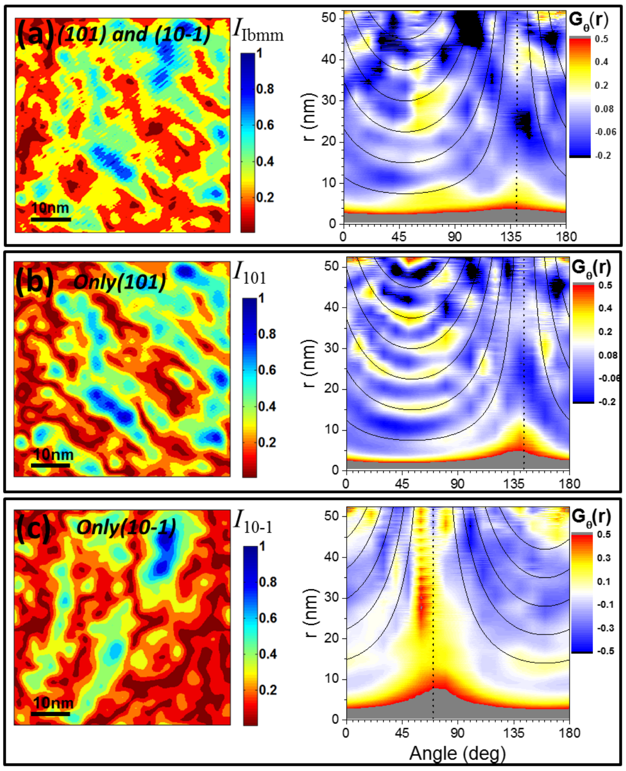

Supplementary Figure 8 – **Four-reflections vs. two reflections images for** $\boldsymbol{x}$ **=0.24 samples**. Variations in the filtered-and-reconstructed HRTEM images by using four or two *Ibmm* reflections, and their respective angle-dependent correlation functions, for a sample with bismuth concentration of x=0.24. **(a)** Same image than in Fig. 4(b) or fig 2(a), obtained by performing the inverse Fourier transform of the four *Ibmm* reflections, $\{101\}_{\mathrm{T}}$ (formed by the $(101)_{\mathrm{T}}$ and $(10\bar{1})_{\mathrm{T}}$ peaks). **(b)** filtered-and-reconstructed image corresponding to the same HRTEM image, but now obtained by only using a set of two reflections corresponding to $(101)_{\mathrm{T}}$ and **(c)** $(10\bar{1})_{\mathrm{T}}$. For all of this images, a resolution reduction from 0.51Å per pixel, to 4.1Å per pixel was performed, in order to average-out the atomic scale information. The angle dependent correlation function for each image is shown in the right hand panels, from which the stripe-like angle evolution can be recognized better in the reconstruction using one set of reflections than with the other. The reconstruction resulting from all the four reflections is a combination of the reconstructions of the individual sets of reflections.

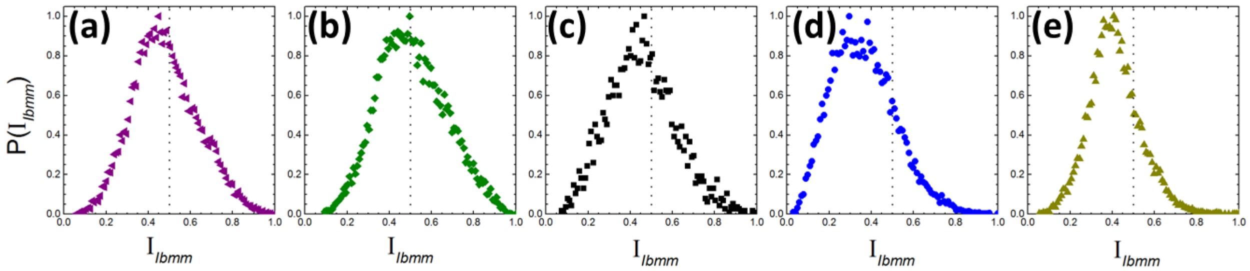

Supplementary Figure 9 – **Probability distribution of orthorhombicity for x =0.18 samples**. Probability distribution of orthorhombicity, $P(I_{Ibmm})$, as a function of the orthorhombicity intensity, I_{Ibmm}, for their corresponding images in figure 1 (with Bi composition of x =0.18).

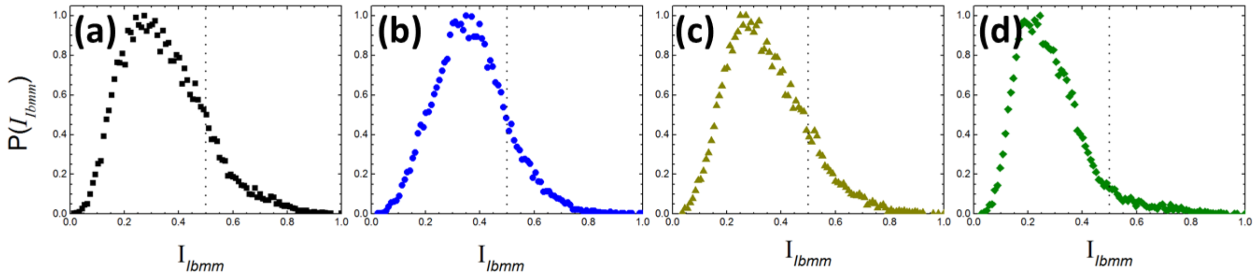

Supplementary Figure 10 – **Probability distribution of orthorhombicity for x =0.24 samples**. Probability distribution of orthorhombicity, $P(I_{Ibmm})$, as a function of the orthorhombicity intensity, I_{Ibmm}, for their corresponding images in figure 2 (with Bi composition of x =0.24).

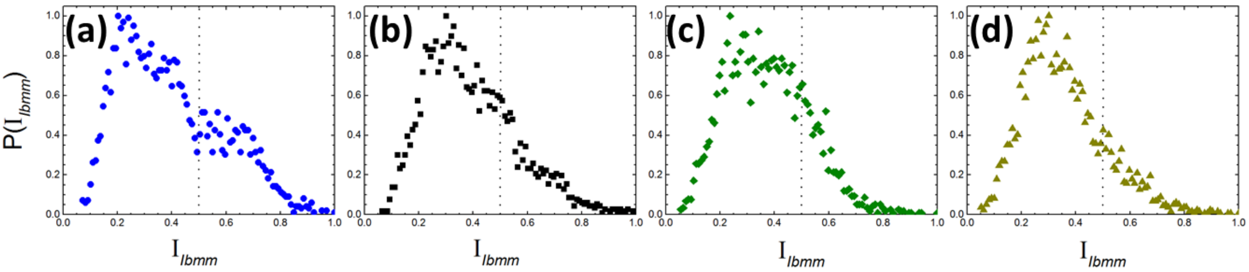

Supplementary Figure 11 – **Probability distribution of orthorhombicity for x =0.28 samples**. Probability distribution of orthorhombicity, $P(I_{Ibmm})$, as a function of the orthorhombicity intensity, I_{Ibmm}, for their corresponding images in figure 3 (with Bi composition of x =0.28).

vspace-0.5cm

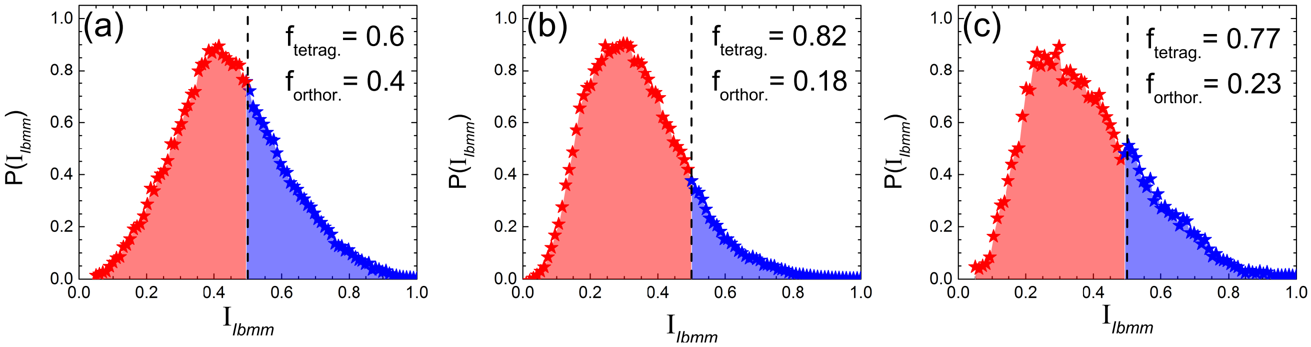

Supplementary Figure 12 – **Average Probability distribution of orthorhombicity** for samples with Bismuth concentration of **(a)** x =0.18, **(b)** x =0.24 and **(c)** x =0.28. The blue and red shadowed regions separate the areas above and below half the intensity, from which the orthorhombic and tetragonal filling fractions can be estimated.

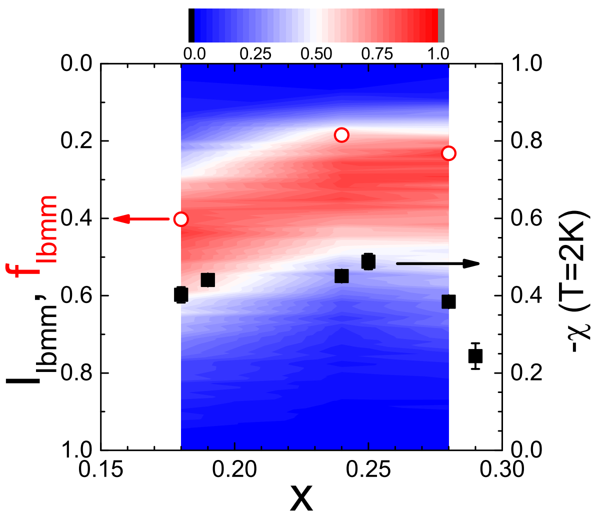

Supplementary Figure 13 – **Evolution of the probability distribution of orthorhombicity**, the orthorhombic fraction and the superconducting volume fraction with Bi doping, in $BaPb_{1-x}Bi_xO_3$. The color scale represents the probability distribution of orthorhombicity, as a function of the orthorhombicity intensity (left scale) and Bi concentration (bottom scale). The red-open circles represent the orthorhombic filling fraction f_{Ibmm} (left scale), calculated from integration of the probability distributions, with a 0.5 criteria. Black squares represent the superconducting volume fraction (right scale) determined from magnetic susceptibility measurements.

SUPPLEMENTARY TABLES

Atom	Wyck.	Site	x/a	y/b	z/c
Ba	4b	-42m	0	0.5	0.25
Pb/Bi	4c	4/m	0	0	0
O1	8h	m.2m	0.2179(14)	0.7179(14)	0
O2	4a	422	0	0	0.25

Supplementary Table 1 – Atomic parameters for tetragonal $BaPb_{1-x}Bi_xO_3$ (space group *I4/mcm*, No. 140), with $x \approx 0.28$, as reported in ref. 1. The site 4c is fully occupied by Pb/Bi, and the Pb to Bi ratio is determined by x.

Atom	Wyck.	Site	x/a	y/b	z/c
Ba	4e	mm2	0.496	0	0.25
Pb/Bi	4a	2/m	0	0	0
O1	4e	mm2	0.0496	0	0.25
O2	8g	.2.	0.25	0.25	0.9741

Supplementary Table 2 – Atomic parameters for orthorhombic $BaPb_{1-x}Bi_xO_3$ (space group *Ibmm*, No. 74), with $x \approx 0.28$, as reported in ref. 1. The site 4a is fully occupied by Pb/Bi, and the Pb to Bi ratio is determined by x.

SUPPLEMENTARY NOTES

Supplementary Note 1. Glazer's notation

The ideal cubic perovskite ABO_3, described by the space group $Pm\bar{3}m$, can be represented as a network of corner-sharing BO_6 octahedra. 'A' atoms sit in the geometric center of the gap between oxygen octahedra. This structure is a "simple" and highly symmetric one; however, most materials with perovskite structures are not in their ideal cubic form, but their structure can nevertheless be represented as coming from distortions from this ideal configuration. The types of distortions found in perovskites can be narrowed down to three types: B-cation displacements within an octahedra; distortions of the BO_6 octahedral unit; and, the most common one and subject of this section, and of Glazer's study [2], the rigid tilting of the corner-sharing BO_6 linked-octahedra units. This last type of distortion was described by Glazer in terms of tilt components along the three different pseudocubic (PC) axes, referred to the original undistorted cubic perovskite. Such pseudocubic axes coincide with the tetrad axes of the octahedra. Given the octahedra corner connections, a tilt about a pseudocubic axis determines the tilts in the directions perpendicular to this axis. However, the tilt of the successive octahedra along the same axis can be either in the same direction or in the opposite direction. With this in mind, the different possibilities of tilt-distortions can be labeled by the notation $a^*b^*c^*$, where a, b, c refer to tilts around the $[100]_{\mathrm{PC}}$, $[010]_{\mathrm{PC}}$ and $[001]_{\mathrm{PC}}$ axes, respectively. If letters are repeated, the tilts are equal for their respective axis. The superscript $*$ can be either 0, for no-tilt along an axis; $+$, for tilt of successive octahedra in the same sense; or $-$, for tilt of successive octahedra in the opposite sense [3]. For example, the $I4/mcm$ space group is represented by the notation $a^0a^0c^-$, which means zero tilt about the $[100]_{\mathrm{PC}}$ and $[010]_{\mathrm{PC}}$ axes, and finite tilt about the $[001]_{\mathrm{PC}}$ axis, with opposite rotation of the successive octahedra along this axis. The $Ibmm$ space group is represented by the notation $a^-a^-c^0$, which means equal tilts about the $[100]_{\mathrm{PC}}$ and $[010]_{\mathrm{PC}}$ axes (equivalent to a tilt about the $[110]_{\mathrm{PC}}$ direction), with opposite rotation of the successive octahedra along these axes, and zero tilt about the $[001]_{\mathrm{PC}}$ axis.

Supplementary Note 2. Electron diffraction patterns

Simulated electron diffraction patterns for tetragonal $I4/mcm$ and orthorhombic $Ibmm$ polymorphs of $BaPb_{1-x}Bi_xO_3$ along the $[001]_{\mathrm{T}}$ and $[010]_{\mathrm{T}}$ zone axis were obtained through the University of Illinois web-based electron microscopy application software (WEB-EMAPS) [4], using the atomic parameters shown in Supplementary Tables 1 and 2. Along both of these zone axis, the (hkl) set of reflections with hlk even, are common to both, orthorhombic and tetragonal phases. However, for both zone axes, the (hkl) set of reflections with hlk odd, appears only in the orthorhombic phase and not in the tetragonal. In ref. 5 we showed that it is possible to recreate the spatial separation of the two polymorphs by systematically masking these diffraction peaks and performing an inverse Fourier transform (IFFT). The result of applying a mask such that only the even (hkl) peaks, common to both the tetragonal and orthorhombic phases, show an ordered array of planes of atoms (see figure 6 in ref. 5). In contrast, the result of applying a mask to the $\{101\}_{\mathrm{T}}/\{110\}_{\mathrm{T}}$ peaks, attributed only to the $Ibmm$ orthorhombic phase, reveals a spatial variation due to the densely intergrown nanostructure, as shown in Fig. 4 of the main manuscript, and in Supplementary Figures 1, 2 and 3.

Supplementary Note 3. Definition of the correlation function

The spatial autocorrelation function $G(\mathbf{r})$ of an image is defined as the statistical correlation of two points separated by a vector $\mathbf{r} = \mathbf{r}_i - \mathbf{r}_j$, where $\mathbf{r}_i$ and $\mathbf{r}_j$ are the positions of those two points in the image [6].

$$G(\mathbf{r}) = \frac{1}{N(\mathbf{r})} \sum_{i,j} \frac{(I_i - \langle I \rangle_1)(I_j - \langle I \rangle_2)}{\sigma_1 \sigma_2} \tag{1}$$

where

$$N(\mathbf{r}) = \sum_{i,j} \delta_{\mathbf{r},(\mathbf{r}_i - \mathbf{r}_j)} \tag{2a}$$

$$\langle I \rangle_1 = \frac{1}{N(\mathbf{r})} \sum_{i,j} \delta_{\mathbf{r},(\mathbf{r}_i - \mathbf{r}_j)} I_i \tag{2b}$$

$$\langle I \rangle_2 = \frac{1}{N(\mathbf{r})} \sum_{i,j} \delta_{\mathbf{r},(\mathbf{r}_i - \mathbf{r}_j)} I_j \tag{2c}$$

$$\sigma_1^2 = \left(\frac{1}{N(\mathbf{r})} \sum_{i,j} \delta_{\mathbf{r},(\mathbf{r}_i - \mathbf{r}_j)} I_i^2 \right) - (\langle I \rangle_1)^2 \tag{2d}$$

$$\sigma_2^2 = \left(\frac{1}{N(\mathbf{r})} \sum_{i,j} \delta_{\mathbf{r},(\mathbf{r}_i - \mathbf{r}_j)} I_j^2 \right) - (\langle I \rangle_2)^2 \tag{2e}$$

The average spatial autocorrelation function $\langle G(\mathbf{r}) \rangle$ is the result of averaging the correlation function of all vectors with the same magnitude $|\mathbf{r}|$. The angle-dependent autocorrelation function $\langle G_\theta(\mathbf{r}) \rangle$ is the result of averaging the correlation function of all vectors with orientation θ with respect to the horizontal axis, and magnitude $|\mathbf{r}|$.

Supplementary Note 4. Correlation function for all the images studied

Supplementary Figures 1, 2 and 3 show $\{110\}_T/\{101\}_T$ filtered-and-reconstructed images (left-hand panels) for a total of five $x = 0.18$ samples (Supplementary Figure 1), four $x = 0.24$ samples (Supplementary Figure 2) and four $x = 0.28$ samples (Supplementary Figure 3), as well as their respective average spatial correlation function $\langle G(\mathbf{r}) \rangle$ (central panels) and angle-dependent spatial correlation function $\langle G_\theta(\mathbf{r}) \rangle$ (right-hand panels). The first image of each figure had been already shown in figure 4 of the main manuscript; however it is shown again for completeness and with the spirit of presenting the average correlation function that was not presented before. The characteristic length scales of phase separation shown in figure 6 of the main manuscript are: (1) stripes periodicity, d, (2) stripes width, w, and (3) the correlation length within a single stripe, ζ. These length scales were determined for all of the figures, and the average value for each quantity determined and plotted as a function of Bi concentration in Fig. 6 of the main manuscript.

Supplementary Note 5. Correlation function for a stripe model

The angular dependent correlation function $\langle G_\theta(\mathbf{r}) \rangle$ was computed for an image of size 128×128 pixels, showing perfect stripes formation, with stripes of width w =14.1 pixels and periodicity d =28.3 pixels, running along an angle α =135° with respect to the horizontal axis (Fig. 5(a) of main manuscript). The color scale of the right hand side plot of Fig. 5(a) represents the value of $\langle G_\theta(\mathbf{r}) \rangle$, as a function of the angle θ with the horizontal axis and the magnitude of $\mathbf{r}$. Maxima of $\langle G_\theta(\mathbf{r}) \rangle$ for this image appear along arcs following the functional form $N * d/\cos((\alpha - 90°) - \theta)$ (shown by the black solid lines), where $N = 1, 2, 3, ...$ Figures 5(b)-(d) of the main manuscript show images of the same size and with stripes of the same width and periodicity as in (a), but where a progressively broken-up character has been introduced for each image. For these images, the maxima of $\langle G_\theta(\mathbf{r}) \rangle$ follow in average the same functional form as the original zero-disorder stripe model, but the local maximum value of $\langle G_\theta(\mathbf{r}) \rangle$ progressively decreases in value, from 1 for the perfect-stripes image in Fig. 5(a), to about 0.2 for the most broken-up image in Fig. 5(d). At the same time, the arcs where the maximum values of $\langle G_\theta(\mathbf{r}) \rangle$ appear to get progressively more broken-up, although its average functional form is preserved, and its periodicity can be well identified. As can be appreciated from the aforementioned figure, this technique proves to be extremely powerful at identifying two-fold rotational symmetries. The angle dependent correlation functions $\langle G_\theta(\mathbf{r}) \rangle$ observed in the $\{110\}_T/\{101\}_T$ filtered-and-reconstructed images analyzed throughout this article show very similar features to the ones observed in this model of broken-up stripes.

Supplementary Note 6. Orientation of stripes

Supplementary Figure 4 shows the orientation of stripes with respect to the $[200]_{\mathrm{T}}$ axis, for all the different samples studied, as a function of Bi concentration. The uncertainty of this measure is large given the imperfect character of the stripe patterns, however, it can be observed that the average value is close to 30° from the $[200]_{\mathrm{T}}$ axis (29°±22°).

Supplementary Note 7. Derivative of the average correlation functions

The length-scale representing the disorder within a stripe, denoted as ζ, is picked-up more clearly in the average correlation function $\langle G(\mathbf{r})\rangle$, as a kink or change of slope in the low-r tail region. This kink is more precisely seen in the derivative of this quantity, as shown in Supplementary Figure 5 for the different Bi compositions and samples of each compositions studied. Black arrows in these plots show the points determining the value of ζ for each sample. The average value of ζ is plotted in figure 6 of the main manuscript, together with the other length scales of phase separation.

Supplementary Note 8. Filtered-and-reconstructed HRTEM images by 4-and-2-reflections

We have shown how, in order to recreate the structural phase separation and to extract its characteristic length scales, virtual dark field (DF) TEM images were obtained by performing the inverse Fourier transform using all four $\{110\}_{\mathrm{T}}/\{101\}_{\mathrm{T}}$ reflections, allowed only in the orthorhombic phase. However, differences in the reconstruction obtained by doing the same process with only two of those reflections can be observed, and these can be ascribed to subtle tiltings of the local crystal axis away from the average zone axis, as explained in Supplementary Figure 6. These subtle variations in tiltings across an area of a sample are possibly a natural consequence of the minimization of strain in a system that needs to accommodate two different crystal structures. Supplementary Figures 7 and 8 shows the three possible filtered-and-reconstructed images for the $\{110\}_{\mathrm{T}}/\{101\}_{\mathrm{T}}$ *Ibmm* reflections, and their corresponding angle dependent correlation functions, for samples with Bi compositions of $x = 0.18$ and $x = 0.24$, respectively. For both figures, the (a) parts have been obtained by using all the four *Ibmm* reflections ((110) and $(1\bar{1}0)$), and they were already shown in the main paper, in Figs. 4(a,b). Parts (b,c) were obtained using only two of these reflections, (110) or $(1\bar{1}0)$. For both compositions, the stripes pattern seems to be better reconstructed by one of the set of peaks, as can be observed in the correlations functions of Supplementary Figures 7(b) and 8(b). The image obtained by using all the four peaks is a combination of the images of both set of reflections, and the characteristic stripe length scales obtained by using this complete set of reflections or the partial set, are equivalent. Given that the zone axis alignment in the experiment is performed using the whole set of reflections, we consider that the more meaningful way of analyzing the average length scales of phase separation is by considering all the four $\{110\}$ reflections.

Supplementary Note 9. Intensity probability distributions

We also computed the probability distribution of intensities of the $\{101\}_{\mathrm{T}}/\{110\}_{\mathrm{T}}$ filtered-and-reconstructed HRTEM images shown in Fig. 4 of the main text, and in Supplementary Figures 1, 2 and 3 of this document. For each image, the intensity in each pixel, which might be labelled the intensity of "orthorhombicity", was normalized by the maximum intensity in the image, so that for all of the images this quantity goes from 0 to 1. Then, we computed the histogram of intensity, dividing the range of intensity into 100 sections. The vertical scale (counts) of each histogram is then normalized, so that for all the images this scale goes from 0 to 1. These curves are proportional to the probability distribution of intensity. The results for each image studied in this work are shown in Supplementary figure 9 for samples with Bi concentration x =0.18, in Supplementary Figure 10 for samples with x =0.24, and in Supplementary Figure 11 for samples with x =0.28. For each composition, all the normalized histograms were averaged, and the result of this averaging is shown in Supplementary Figure 12. From these averaged histograms we estimated the orthorhombic filling fraction, f_{Ibmm}, as the normalized-integrated area above half the total intensity (as shown in the blue-shadowed areas in Supplementary Figure 12).

The evolution of the orthorhombic volume fraction as a function of Bi concentration can be better visualized in Supplementary Figure 13. The left axis of this figure shows an inverted scale (from 1 to 0) of the orthorhombic intensity. The colors in the contour plot represent values of the probability of "orthorhombicity", i.e., the y scale in the averaged histograms shown in Supplementary Figure 12, as a function of orthorhombic intensity and Bi concentration.

From this figure we can observe that the maximum of the probability distribution of "orthorhombicity" (red colors) is shifted toward lower values of orthorhombicity for optimal doping, compared to samples with lower and higher Bi concentrations. As a consequence, if we represent the orthorhombic volume fraction f_{Ibmm} as the normalized-integrated area above half the total intensity, it is minimum at optimal doping, which means that the tetragonal fraction, $f_{I4/mcm} = 1 - f_{Ibmm}$ is maximum at this composition, with a value of 0.82 ± 0.08. The evolution of the inverse of the orthorhombic fraction, ie., the tetragonal fraction, approximately tracks the evolution of the superconducting volume fraction, shown in the right scale and as black squares. These observations are consistent with results from x-ray and neutron diffraction experiments by E. Climent-Pascual *et al.*[1] in polycrystalline samples, and suggest a direct connection between the tetragonal distortion and superconductivity.

SUPPLEMENTARY REFERENCES

[1] Climent-Pascual, E., Ni, N., Jia, S., Huang, Q., and Cava, R. J. Polymorphism in $BaPb_{1-x}Bi_xO_3$ at the superconducting compositions. *Phys. Rev. B* **83**, 174512 (2011).

[2] Glazer, A. M. The clasification of tilted octahedra in perovskites. *Acta Cryst.* **B28**, 3384–3392 (1972).

[3] Howard, C. J. and Stokes, H. T. Group theoretical analysis of octahedral tilting in perovskites. *Acta Cryst.* **B54**, 782–789 (1998).

[4] Zuo, J. and Mabon, J. C. Web-based electron microscopy application software: Web-emaps. University of Illinois. http://emaps.mrl.uiuc.edu., (1992).

[5] Giraldo-Gallo, P., Lee, H.-O., Zhang, Y., Kramer, M. J., Beasley, M. R., Geballe, T. H., and Fisher, I. R. Field-tuned superconductor-insulator transition in $BaPb_{1-x}Bi_xO_3$. *Phys. Rev. B* **85**, 174503 (2012).

[6] Fratini, M., Poccia, N., Ricci, A., Campi, G., Burghammer, M., Aeppli, G., and Bianconi, A. Scale-free structural organization of oxygen interstitials in La_2CuO_{4+y}. *Nature* **466**, 841–844 (2010).
